# Effects of opioid-free anaesthesia on postoperative nausea and vomiting in patients undergoing video-assisted thoracoscopic surgery (OFA-PONV trial): study protocol for a randomised controlled trial

**DOI:** 10.1186/s13063-023-07859-z

**Published:** 2023-12-20

**Authors:** Xiang Yan, Chen Liang, Jia Jiang, Ying Ji, Anshi Wu, Changwei Wei

**Affiliations:** 1 Department of Anaesthesiology, Beijing ChaoYang Hospital, Capital Medical University, No. 8 Gongren Tiyuchang Nanlu, Chaoyang District, Beijing, China; 2 Department of Medical Statistics, Medieco Group Co., Ltd, Beijing, China; 3 Department of Thoracic Surgery, Beijing ChaoYang Hospital, Capital Medical University, Beijing, China

**Keywords:** Anaesthesia, Opioid, Nausea, Vomiting, Video-assisted, Surgery

## Abstract

**Background:**

Postoperative nausea and vomiting (PONV) is a common complication after general anaesthesia and is associated with morbidity and prolonged length of stay. Growing evidence suggest that opioid-free general anaesthesia (OFA) may reduce PONV in various surgical settings. We aim to evaluate the efficacy of OFA on the incidence of PONV compared with opioid-based anaesthesia among adults undergoing thoracoscopic surgery.

**Methods:**

This is a prospective, single-centre, randomised controlled trial comparing OFA and opioid-based anaesthesia for thoracoscopic surgery. A total of 168 adults will be randomised with a 1:1 ratio to receive either opioid-free anaesthesia or opioid-based anaesthesia. The primary outcome will be the incidence of PONV within 24 h after operation. The secondary outcomes will include the severity of PONV, quality of recovery, pain at rest, 6-min walking test, and health-related quality of life after operation.

**Discussion:**

The benefit-risk of OFA for patients after operation is contradictory in previous studies, so further study is required. This trial will focus on the effect of OFA on the incidence of PONV in patients undergoing thoracoscopic surgery. This trial adopts uniformed PONV and perioperative pain management, standardised randomised and blind, clear-cut inclusion and exclusion criteria, and standardised scales to assess the severity of PONV after surgery, the quality of postoperative recovery, and the health status at 6 months. The findings of this study will help to provide references to promote early recovery of patients after lung surgery.

**Trial registration:**

ClinicalTrials.gov NCT05411159. Registered on 9 June 2022.

## Background

Postoperative nausea and vomiting (PONV) is one of the most common complications after general anaesthesia [1, 2]. It is reported that up to 50% of patients after video-assisted thoracoscopic surgery (VATs) experienced PONV [3, 4], which contributes to aspiration, bleeding, insomnia, poor appetite, dissatisfaction, delayed recovery, and discharge after surgery [5–10]. Risk factors of PONV include younger age, female gender, history of PONV, history of motion sickness, non-smoker, and the use of volatile anaesthetics and opioids [2, 5]. Opioids contribute to PONV by stimulating the opioid receptors located in the chemoreceptor trigger zone of the medulla oblongata, vestibular organs, and gastrointestinal tract [11]. Besides the PONV, the use of opioids in the perioperative period is also associated with hypoxemia, itching, dizziness, fatigue, and urinary retention after surgery [12].

Guidelines for enhanced recovery appeal to minimize opioid consumption by multimodal analgesia regimens in patients after lung surgery [13]. Opioid-free general anaesthesia (OFA) might be a choice for that purpose [14, 15]. Meta-analysis showed that the incidence of PONV was decreased by 23–73% in patients with OFA than those with opioid-based anaesthesia after gynaecology, breast, and abdominal surgeries [14–16]. There are few published trials that explored the effect of OFA on PONV among patients undergoing thoracic surgery [4, 17]. In a randomised trial of 97 patients after VATs, the incidence of PONV was found to be significantly lower in the OFA group than that in the opioid-based anaesthesia group (4% vs 42%) [4]. However, no association was observed between OFA and the lower incidence of PONV in a propensity score study of 81 patients after VATs [17]. There were no risk assessment and standard prophylaxis for preventing PONV, which may contribute to the different results of previous studies [4, 17].

Therefore, we design a prospective randomised controlled trial to further evaluate the effect of OFA on the incidence of PONV in patients undergoing VATs. The hypothesis is that the OFA could reduce the incidence of PONV compared with the opioid-based anaesthesia among these patients.

## Methods

### Study design

**Fig. 1:**
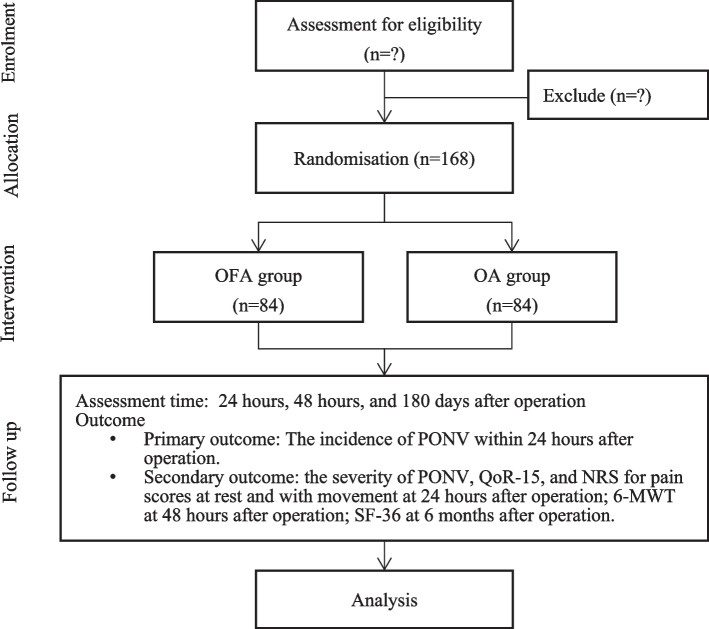
Flow diagram for OFA-PONV trial. OFA, opioid-free anaesthesia; OA, opioid-based anaesthesia; PONV, postoperative nausea and vomiting; QoR-15, quality of recovery-15 scale; NRS, numerical rating scale; 6-MWT, 6-minute walk test; SF-36, 36-item short-form survey

This prospective, single-centre, randomised controlled trial with two parallel arms was designed to examine whether OFA will reduce the incidence of PONV in patients undergoing VATs. Inpatients planning to receive elective thoracoscopic lobectomy or wedge resection under general anaesthesia will be recruited at Beijing Chaoyang Hospital, Capital Medical University, from June 2022 to December 2024. Preoperative interview will be conducted by specially trained research assistants. They will inform patients about the study objectives, risks, and benefits and obtain written informed consent from participants. Figure 1 shows the design of the study.

The protocol was prepared according to the Declaration of Helsinki principles, Standard Protocol Items: Recommendations for Interventional Trials (SPIRIT) [18], and registered in www.clincaltrials.gov (Trial registration number: NCT 05411159).

### Study population

#### Inclusion criteria

On the day before surgery, patients will be screened by a face-to-face visit according to the eligibility criteria. The inclusion criteria include (1) adult patients aged 18–65 years with preoperative pulmonary computerized tomography (CT) diagnosis of lung space-occupying lesions and (2) patients who plan to undergo elective thoracoscopic lobectomy or wedge resection under general anaesthesia.

#### Exclusion criteria

Patients will be excluded if they have any of the following exclusion criteria: (1) American Society of Anesthesiologists (ASA) physical status > IV; (2) body mass index > 35 kg/m^2^; (3) unable to communicate before surgery; (4) receive radiation therapy, chemotherapy, opioids, or hormonal drugs within 14 days before surgery; (5) known to be intolerant of the anaesthesia protocol of this study; (6) expected to experience the prolonged length of mechanical ventilation usage after surgery; (7) decline to participate in the study.

### Randomisation and blinding

Eligible patients will be randomised to receive opioid-free anaesthesia (OFA group) or opioid-based anaesthesia (OA group) with an allocation ratio of 1:1 after providing written informed consent. The randomisation sequence (block size 4 or 6) will be generated by an independent researcher using SPSS version 25.0 (IBM SPSS, Chicago, IL). Then, the randomisation codes will be placed sequentially in consecutively numbered, opaque, sealed, stapled envelopes. After a participant enters the operating room, the attending anaesthesiologist will open the corresponding numbered envelope, confirm the patient’s assignment, and administer the assigned anaesthesia protocol.

The outcome assessors, thoracic surgeons, and nursing stuff will be blinded to the group assignment, unless research-related serious adverse events happen. Enrolled participants and their legal representatives will also be blinded to the received anaesthesia method.

### Standard anaesthesia management

Standard anaesthesia will be administered to maintain the comparability between groups other than the intervention.

Intraoperative monitoring will include electrocardiogram (ECG), peripheral oxygen saturation, invasive blood pressure, end-tidal carbon dioxide, and processed electroencephalogram (EEG). The depth of sedation during surgery was monitored by the wavelet index (WLi) and pain threshold index (PTi); they are calculated from changes in EEG signals by multifunction combination monitor HXD-I (Heilongjiang Huaxiang Technology Co., Ltd., Heilongjiang, China) [4]. The WLi ranges from 0 to 100, with 0 indicating the disappearance of EEG while 100 indicating awake status. The intraoperative dosage of anaesthetic will be adjusted to keep the WLi between 40 and 60. The PTi could be used to monitor the integrated brain response to anti-nociceptive stimuli during general anaesthesia. The score of PTi ranges from 0 to 100, with a higher score indicating a stronger stimulus. In addition, radial arterial blood gas will be measured before and after one-lung ventilation.

After a patient enters the operating room, atropine (0.25 mg) will be given intravenously. Anaesthesia will be induced with intravenous propofol (2–3 mg/kg), rocuronium (0.6–0.8 mg/kg). During the operation, anaesthesia will be administered using desflurane at 0.5 to 1 minimum alveolar concentration. Continuous or intermittent additions of propofol or rocuronium were allowed throughout the study to modulate the intraoperative depth of sedation or muscle relaxation.

All patients will receive bronchial intubation during the VATs. The mechanical ventilation will be performed as follows. After intubation, the inspired oxygen concentration, tidal volume, and respiratory rate will be 100%, 6–8 ml/kg, and 12–18/min, respectively, while the tidal volume and respiratory rate will be set to 4–6 ml/kg and 12–20/min to maintain normocapnia during lung isolation. Intraoperative mean arterial pressure will be maintained within ± 25% of the baseline value. The vasoactive drugs can be used as needed.

After surgery, the endotracheal tube will be removed when the arterial oxygen saturation under spontaneous rhythm breathing is maintained above 90%. The patient is then transferred to the postoperative anaesthesia care unit (PACU). After the modified Aldrete score [19] reaches 9, the patient will be allowed to return to the ward.

Thereafter, each patient will plan to take ibuprofen 0.2 g orally every 8 h and receive intravenous infusion of sufentanil (2 µg/h) until 48 h after surgery. During this period, the sufentanil infusion could be turned off if the participant experience any one of the opioid-related complications (described below). Available rescue analgesics in the thoracic ward include intravenous nonsteroidal anti-inflammatory agents (NSAIDs) and tramadol.

Referring to the recommendations of the guidelines, and considering the feasibility in our clinical practice, we will adopt 3 regimens to reduce the incidence of PONV in patients, that is, intravenous dexamethasone, use propofol for induction, and receive regional block to minimize intraoperative opioids requirement [2]. Due to the local health insurance policies, 5-HT_3_ receptor antagonists and metoclopramide will be used as a treatment for PONV other than as prophylactic drugs in the ward.

### Interventions and intraoperative pain management

Patients will be randomly assigned to the OFA group or OA group. All patients will receive intravenous lidocaine (1.5 mg/kg) and flurbiprofen (50 mg) before induction as part of multimodal analgesia regimens. Another dose of flurbiprofen (50 mg) will be administered at the end of surgery. Before incision, an ultrasound-guided thoracic paravertebral block (0.5% ropivacaine 20 ml) will be performed between the fourth and fifth thoracic vertebrae on the surgical side.

#### OFA group

Patients will receive opioid-free general anaesthesia during VATs according to the regimens reported in previous studies [4, 17]. Before anaesthesia induction, dexmedetomidine (0.5 ug/kg for 15 min) will be infused intravenously. Then, dexmedetomidine (0.5 ug/kg/h), and lidocaine (1.5 mg/kg/h) will be administered intravenously until the end of VATs.

#### OA group

Patients will receive opioid-based anaesthesia during VATs. Sufentanil (0.3–0.4 ug/kg) will be administered for induction, and then remifentanil (0.1–0.2 ug/kg/min) will be given until the end of VATs.

### Reporting of adverse events

After participants were included in the study, the occurrence of adverse events and complications will be recorded, and the severity will be assessed according to the ClassIntra [20] and Clavien-Dindo [21] surgical complication categories. In this study, the expected anaesthesia-related adverse events will include cardiac arrest, local anaesthetic toxicity, nerve block puncture site hematoma, hypoxemia, hypotension, and delayed awakening.

Any specific reasons for violation of the study protocol will be recorded. The attending anaesthesiologist could not comply with the standard study protocol if any of the following circumstances occurs during operation.

1. The participant’s blood pressure is hard to maintain within ± 25% of the baseline level.
2. The participant’s heart rate is lower than 40 times/min (even being administered with atropine intravenously).
3. The participant’s peripheral pulse saturation becomes less than 90% and lasts for more than 5 min.
4. Other intraoperative emergencies judged by the attending anaesthesiologist.

### Data collection and measurement

At baseline, the demographic characteristics, medical history, and laboratory tests will be obtained. All participants will receive lung CT, pulmonary function test, 6-minute walk test (6-MWT), and Apfel’s PONV risk assessment [1]. Karnofsky Performance Status (KPS), ASA physical status classification, and Charlson Comorbidity Index will also be used to assess the functional status of participants. During the operation, the intraoperative medications and surgical and anaesthetic characteristics will be recorded by anaesthesiologist with the predesigned case report form (CRF). The detailed information will include the site and type of surgery resection, physiological parameters, EEG (including WLi and PTi), the doses of anaesthetics, arterial blood gas results, estimated blood loss, blood transfusion, duration of surgery, and anaesthesia. Intraoperative complications and adverse events will also be recorded. In PACU, the outcome assessors will assess and record the occurrence of PONV, pain scores, sedation status (Richmond Agitation-Sedation score), medications, complications, and the duration of PACU stay after VATs.

During the early postoperative period (before discharge), outcome assessors will conduct daily face-to-face interviews with each participant. PONV and pain will assessed on the first and second day. The severity of PONV and time to the first PONV will be ascertained according to the Simplified PONV impact scale [9]. Numerical Rating Scale (NRS) will be used to assess pain at rest and with movement (0–10 score, higher score represents worse). The quality of recovery on the first day will be assessed using the quality of recovery-15 (QoR-15) scale (0–150 score, higher score represents better) [22]. The 6-MWT (farther distance is better) will be tested on the second day after surgery [23]. Time to the first drinking, eating, and postoperative out-of-bed mobilization will be recorded in CRF. At discharge (usually 3 days after operation), KPS and total costs will be recorded. Long-term follow-up will be conducted through telephone or video to collect information 6 months after operation. For example, the 36-item short-form survey (SF-36) will be used to assess the health and well-being status of participants (higher score represents a more favourable health state). Postoperative opioid-related complications (hypoxemia, hypotension, bradycardia, itching, tachycardia, dizziness, fatigue, urinary retention, and constipation) and all-cause mortality will also be recorded at each interview after operation. Table 1 presents the schedule of patient enrolment, allocation, and outcome measurements.

**Table 1:** Schedule of enrolment, intervention, and assessment

Time point	Study period						
	**Enrolment**	**Post-allocation**		**Follow-up**			
	**Preoperation**	**Intraoperative**	**Postoperative anaesthesia care unit**	**24 h after operation**	**48 h after operation**	**discharge (3 days after operation)**	**6 months after operation**
**Enrolment**							
Eligibility screen	√						
Informed consent	√						
**Assessment**							
Baseline variables	√						
Lung imaging	√						
Apfel’s PONV risk assessment	√						
Karnofsky Performance Status	√					√	
Intraoperative data		√					
EEG monitoring		√					
Blood gas		√					
**PONV**^**a**^			√	√	√		
Complications and adverse events		√	√	√	√	√	√
Qor-15				√			
Pain at rest and with movement				√	√		
6-MWT	√				√		
SF-36							√

According to SPIRIT statement of defining standard protocol items for clinical trials

*PONV* postoperative nausea and vomiting, *QoR-15* quality of recovery-15 scale, *6-MWT* 6-minute walk test, *SF-36* 36-item short-form survey

^a^Primary outcome

### Primary outcome

The primary outcome is the cumulative incidence of PONV within 24 h after operation, which will be assessed by a blinded outcome assessor using Myles’s simplified PONV impact scale (score > 0 is regarded as the occurrence of PONV) [9]. This scale consists of vomited or dry-retching (0–3 score) and nausea (0–3 score), with higher scores indicating being more severe.

### Secondary outcomes

- ➢ The severity of PONV during 24 h after surgery, scored by Myles’s simplified PONV impact scale [9]
- ➢ The score of QoR-15 at 24 h after surgery.
- ➢ The NRS scores of pain at rest and with movement at 24 h after surgery
- ➢ The 6-MWT at 48 h after surgery
- ➢ Health and well-being (assessed by SF-36) at 6 months after surgery

### Other pre-specified outcome measures

- ➢ Length of hospital stay, measured by days from surgery to discharge
- ➢ Length of PACU stay, measured by minutes
- ➢ Intraoperative complications assessed according to the ClassIntra complication classifications [20]
- ➢ Postoperative complications assessed according to the Clavien-Dindo surgical complication classifications within 7 days after surgery or discharge [21]

### Data monitoring committee

The project will be monitored by the Data Monitoring Committee composed of specialists in anaesthesiology, thoracic surgery, ethics, statistics, and methodology. They will audit through regular interviews or telephone calls and be responsible for terminating the research in case of severe adverse events.

### Statistical analysis

All outcomes will be analysed according to the statistical analysis plan with SPSS version 25.0 or above (IBM SPSS, Chicago, IL) or SAS version 9.4 or above (SAS Institute). The primary analysis will be conducted in the modified intention-to-treat (mITT) population including all patients who were randomized and finished VATs under general anaesthesia. In addition, we will take the per-protocol set as the secondary set for the primary outcome.

Normally distributed continuous variables will be described by mean ± standard deviation and compared by Student’s *t*-test, while skewed distributed continuous variables will be described by median [interquartile range (IQR)] and compared by Mann–Whitney *U* test. Categorical variables will be reported as frequency (percentage) and compared by Pearson’s chi-squared test or Fisher’s exact test.

Univariate log-binomial model will be applied to evaluate the effect of OFA on the primary outcome. The result will be presented as relative risk (RR) with 95% confidence interval (CI). To further explore the effect of the intervention on the incidence of PONV, multivariate log-binomial model adjusting for either imbalanced baseline characteristics or potential confounders (such as age, sex, history of PONV or motion sickness, smoking history, and duration of anaesthesia) will be employed to estimate the adjusted RR and corresponding 95%CI. If the log-binomial model fails to converge, the Poisson regression model with robust variance estimation will be applied instead. We will use methods including Kaplan–Meier curves and log-rank tests to compare the time-to-event variables between two groups.

Prespecified subgroup analysis will be analysed by stratifying the gender, smoker, history of PONV or motion sickness, and duration of anaesthesia. No imputation will be performed for missing data.

The statistical significance will be defined as the two-sided *P*-value less than 0.05.

### Sample size calculation

The sample size calculation is based on the primary outcome. We assume that the cumulative incidence of PONV after VATs will be 40% according to that reported in previous publications (ranged from 28.6 to 41.7% in adults during the first day after VATs [4, 8, 24]). Assuming a 50% reduction of the cumulative incidence of PONV in the OFA group compared to the OA group [16], a sample size of 84 for each group (168 in total) will be required to achieve a power of 80% to detect the difference at a two-side *α* level of 0.05 with a dropout rate of 5%.

## Discussion

This trial will investigate whether OFA could reduce the incidence of PONV compared with opioid-based general anaesthesia in patients undergoing VATs.

The benefit-risk of OFA for patients after operation is contradictory in previous studies [15, 16], so further study is required. Differences in the results of those studies may be related to the different surgical populations or the different regimens of OFA, so there is no consensus on the optimal choice. The OFA regimen is the use of a combination of non-opioid analgesics to achieve anti-nociception during surgery [25–29]. An et al. [4] performed the OFA regimen using dexmedetomidine, ketorolac, sevoflurane, and regional block in patients undergoing VATs. The results showed that OFA could provide a comparable analgesia compared with opioid-based anaesthesia during VATs [4]. In the study of Selim et al. [17], the OFA was achieved by using dexmedetomidine, ketamine, lidocaine, sevoflurane, and regional block. OFA was found to be associated with less postoperative morphine consumption (medians = 28.5 mg vs. 55.0 mg, *P*-value = 0.002) and lower pain score (medians = 0 vs. 2.5, *P*-value = 0.034) at 48 h compared with opioid-based anaesthesia. Refer to previous studies, we will adopt an OFA regimen including dexmedetomidine, NSAIDs, lidocaine, desflurane, and regional block during VATs and suppose that its analgesic effect would be similar to that in the OA group.

PONV may be transient or mild, but the impact on patients can be much more severe, including restricted oral intake, inability to mobilize after surgery, and delayed recovery after surgery [2]. The intensity, pattern, and duration of nausea are all important for the selection of prevention and treatment options for PONV [9]. But the impact of PONV on the quality of recovery in patients after thoracic surgery was not available in previous trials [4, 17]. Therefore, in this trial, we will select a validated simplified scale to quantify the clinical severity of PONV in patients after VATs [9]. We suspect that a reduction in the incidence or severity of PONV may improve the quality of recovery after surgery.

Compared with opioid-based anaesthesia regimens, OFA provides a comparable analgesic effect during VATs. Meanwhile, it may have the potential to save on opioid consumption after lung surgery. An et al. compared the effects of OFA with opioid-based anaesthesia on intraoperative pain control (assessed by EEG) in a randomised trial (*n* = 97) and found that they were comparable in patients during VATs [4]. Selim et al. found patients with OFA are associated with lower cumulative morphine consumption and pain scores at 48 h after VATs compared to those with opioid-based anaesthesia [17]. And there is no direct evidence that OFA is associated with the increased risk of severe pain, postoperative complications, or adverse events [14, 16].

In conclusion, this study will provide clinical evidence on the potential benefit of reducing the incidence of PONV by the OFA among adult patients undergoing VATs and may thus offer an option for those patients with high risk of PONV.

## Trial status

The randomised trial, which commenced in June 2022, is currently in the phase of participant enrolment. The current protocol is version 1.1.3 (issue date 30 June 2022). The estimated completion date is May 2024.

## Data Availability

Available upon request from the corresponding author (CWW) at changwei.wei@ccmu.edu.cn.

## Abbreviations

6-MWT: 6-minute walk test
ASA: American Society of Anesthesiologists
CI: Confidence interval
CRF: Case report form
CT: Computerized tomography
ECG: Electrocardiogram
EEG: Electroencephalogram
IQR: Interquartile range
KPS: Karnofsky Performance Status
mITT: Modified intention-to-treat
NRS: Numerical rating scale
NSAIDs: Nonsteroidal anti-inflammatory agents
OFA: Opioid-free general anaesthesia
PACU: Postoperative anaesthesia care unit
PONV: Postoperative nausea and vomiting
PTi: Pain threshold index
Qor-15: Quality of recovery-15 scale
RR: Relative risk
VATs: Video-assisted thoracoscopic surgery
WLi: Wavelet index
